# Protocol for a systematic review and thematic synthesis of patient experiences of central venous access devices in anti-cancer treatment

**DOI:** 10.1186/s13643-018-0721-x

**Published:** 2018-04-18

**Authors:** Caoimhe Ryan, Hannah Hesselgreaves, Olivia Wu, Jim Paul, Judith Dixon-Hughes, Jonathan G. Moss

**Affiliations:** 1 Institute of Health and Wellbeing, University of Glasgow, Glasgow, UK; 2 School of Medical Education, Newcastle University, Newcastle upon Tyne, UK; 3 Institute of Cancer Sciences, University of Glasgow, Glasgow, UK; 4 Gartnavel General Hospital, Glasgow, UK

**Keywords:** Systematic review, Protocol, Central venous access devices, Vascular access, Chemotherapy, Patient experience, Patient perspectives

## Abstract

**Background:**

Three types of central venous access devices (CVADs)—peripherally inserted central catheters (PICCs), skin-tunnelled central catheters (Hickman-type devices), and implantable chest wall Ports (Ports)—are routinely used in the intravenous administration of anti-cancer treatment. These devices avoid the need for peripheral cannulation and allow for home delivery of treatment. Assessments of these devices have tended to focus on medical and economic factors, but there is increased interest in the importance of patient experiences and perspectives in this area. The aim of this systematic review is to synthesise existing research regarding patient experiences of these CVADs to help clinicians guide, prepare, and support patients receiving CVADs for the administration of anti-cancer treatment.

**Method:**

A systematic search of MEDLINE, Embase, and CINAHL research databases will be carried out along with a supplementary reference list search. This review will include quantitative, qualitative, and mixed methods studies published in peer-review journals, reporting some aspect(s) of patient experiences or perspectives regarding the use of PICC, Hickman, or Port CVADs for the administration of anti-cancer drugs. The methodological quality and risk of bias of included papers will be assessed using the Mixed Methods Appraisal Tool (MMAT). Relevant outcome data will be extracted from included studies and analysed using a thematic synthesis approach.

**Discussion:**

The results section of the review will comprise thematic synthesis of quantitative studies, thematic synthesis of qualitative studies, and the aggregation of the two. Results will aim to offer an account of current understandings of patient experiences and perspective regarding PICC, Hickman-type, and Port devices in the context of anti-cancer treatment. Confidence in cumulative evidence will be assessed using the Confidence in the Evidence from Reviews of Qualitative research (CERQual) approach.

**Systematic review registration:**

This systematic review protocol is registered with the International Prospective Register of Systematic Reviews (PROSPERO). Registration number: CRD42017065851. This protocol was prepared using the Preferred Reporting Items for Systematic Reviews and Meta-Analyses for Protocols checklist (PRISMA-P) (Shamseer et al., BMJ 349: 2015).

**Electronic supplementary material:**

The online version of this article (10.1186/s13643-018-0721-x) contains supplementary material, which is available to authorized users.

## Background

Three types of central venous access devices (CVADs) are routinely used in the intravenous administration of anti-cancer treatment: peripherally inserted central catheters (PICCs), skin-tunnelled central catheters (Hickman-type devices), and implantable chest wall Ports (Ports).

All three deliver the drug into the superior vena cava, which drains directly into the right atrium of the heart. PICC lines are inserted into a peripheral vein in the arm. The end of the line remains outside the body, emerging from a point usually above the elbow. Hickman-type devices are inserted into a central vein in the neck or upper chest. The end of the line is tunnelled under the skin to emerge from the chest, where it sits outside the body. Chest wall Ports constitute a closed-system that is fully implanted in the body with no external lines. Vascular access is via needle puncture.

The main benefit of the devices is that they avoid the need for peripheral cannulation which relies on peripheral arm veins that easily become occluded. Moreover, peripheral cannulation usually involves repeated needle insertions which patients tend to find unpleasant, even painful and distressing [1]. They also allow for home delivery of treatment via portable chemotherapy infusion pump.

Patient-centred approaches in the area of vascular access exist but are limited. Published reports on the use of vascular access devices in the context of anti-cancer treatment have tended to focus on medical and economic factors such as cost, maintenance, infection, and other complications [2, 3], with much less attention given to patient experiences and perspectives. It is therefore difficult to gain a clear picture of how patients experience PICC, Hickman, and Port devices [4]. Developing a better understanding of these experiences would offer important insight into the roles these devices play in patients’ quality of life and in the burden of treatment. Moreover, a comparison of the effects and experiences of these different devices would be important in guiding device selection where more than one device is clinically suitable.

To our knowledge no systematic review has previously been carried out on this topic. The present review will draw together existing approaches to recording and assessing patient experiences of these CVADs in order to produce a coherent understanding of such experiences. This will help clinicians guide, prepare, and support patients receiving CVADs for the administration of anti-cancer treatment.

### Objectives

The aim of this review is to synthesise existing literature that investigates patient experiences with PICC, Hickman-type, and Port devices in the context of anti-cancer treatment.

This review will address the following questions: What are patients’ experiences and perspectives regarding the three different central venous access devices implanted for delivering long-term anti-cancer treatment: peripherally inserted central catheters (PICC), Hickman-type tunnelled catheters, and totally implanted chest wall Ports? What effect (if any) do these CVADs have on patients’ quality of life in the context of anti-cancer treatment, and what aspects of each device are important in this respect? To what extent is it possible to compare and contrast patient experiences of different CVADs? What are the limitations of the existing literature, and what additional research is needed?

## Method

### Eligibility criteria

This review will include empirical studies published in peer-review journals meeting review-specific eligibility criteria. These criteria were developed in line with the existing search tools PICO (Population, Intervention, Comparison, Outcome) [5] and SPIDER (Sample, Phenomenon of Interest, Design, Evaluation, Research type) [6]. PICO is typically used in systematic reviews of quantitative research, answering clinical questions. SPIDER was adapted from the former for use in systematic reviews of qualitative and mixed method research. As our review will comprise quantitative, qualitative, and mixed methods studies, our eligibility criteria include elements from both.

As the use of PICCs and chest wall Ports in cancer treatment was not well established until the 1990s and involved a learning curve of several years, this review will consider only studies published from the year 1997 onward.

#### Participant population

Studies will be eligible for inclusion where participants are adult or paediatric patients (or parents/guardians of paediatric patients) diagnosed with any type of cancer including solid malignancy and haematologic malignancy, who have received PICC, Hickman-type or chest wall Port CVADs for the primary purpose of the administration of anti-cancer drugs.

#### Intervention

Included studies will involve the implantation (via any technique) of any of the following venous access devices for the long-term administration of any anti-cancer treatment: (i) peripherally inserted central catheters (PICC), (ii) subcutaneously tunnelled central catheters (Hickman-type device), and (iii) implantable chest wall Ports (Port).

#### Comparator/control

Studies will not be included or excluded on the basis of comparator. Where relevant and appropriate, comparisons between devices or other forms of venous access in the administration of anti-cancer treatment will be considered in analysis.

#### Design

The review will include empirical studies using quantitative and/or qualitative analytic methods. It will exclude non-empirical studies but will not limit inclusion based on study design. Studies that report outcomes regarding CVADs but do not report or discuss any patient experience will also be excluded, as will studies that do not provide sufficient detail for a thematic synthesis—i.e. studies that do not contribute to themes or where data cannot be themed.

#### Context

This review will include studies conducted in oncology settings such as hospitals, and clinics, or related contexts including home-treatment, patient care facilities, or patient groups/organisations.

#### Evaluation/outcomes

Finally, to be eligible for inclusion study, participants must have completed a measure (e.g. questionnaire, survey) or provided an account (e.g. interview, focus group) of some aspect of their own experience with or perspectives on CVADs or in the case of paediatric patients, where parents/guardians similarly report on some aspect of their own and their children’s experiences and perspectives (please see ‘Outcomes and Prioritisation’ section, below, for explanations of these outcomes).

### Information sources

A systematic search strategy will be used to search MEDLINE, Embase, and CINAHL research databases. A further important component of our search strategy, especially for the purposes of identifying relevant qualitative research, involves forward and backward citation tracking of publications meeting the inclusion criteria, via Web of Science and Google Scholar. In addition, experts including authors of key papers will be contacted to minimise the likelihood of overlooking key sources.

### Search strategy

Search terms will target three key domains: (i) central venous access devices, (ii) patient population, and (iii) patient experiences. Search terms for Hickman and Port devices and for cancer and chemotherapy will be adapted from an existing systematic review comparing the clinical effectiveness of these devices [7]. The search strategy has been constructed with advice and guidance from an information scientist. An example of search terms and strategy is provided in Appendix.

### Data management

Details of all searches will be recorded. Search results will be downloaded to EndNote desktop software. Studies sourced through supplemental hand searching will be recorded and imported into EndNote.

### Selection process

Duplicate publications will be removed. The titles and abstracts of all remaining publications will be assessed against inclusion criteria. Those not meeting these criteria will be excluded. All titles and abstracts identified at this stage will also be screened independently by a second reviewer. Disparities will be resolved through discussion or in consultation with a third party. Full texts of the remaining publications will be obtained for further review. Reasons for further exclusions at this stage will be documented. The selection process, including search results and reasons for exclusion at each stage of screening, will be recorded and represented in a PRISMA flow diagram (Additional file 1)[8].

### Data collection process

Data extraction will be performed by one reviewer using a data extraction form developed by the researchers for the purposes of this review. This form will be refined by the reviewer until the data extraction is complete, to ensure the appropriateness and usefulness of all fields.

### Data items

For all studies, descriptive data will be extracted regarding type(s) of vascular access device included in the study, patient population, sample size, stated research aims, study context, study design, methodology, reason for measurement of patient experience, and timing of measurement (in relation to device placement).

For quantitative studies, outcome data including descriptive and inferential statistics will be extracted for measures of patient experiences or perspectives regarding CVADs. Details of measurement instruments will also be recorded. The purpose of qualitative synthesis is to ‘go beyond’ primary studies. This means the data to be extracted from the qualitative studies comprises the analyses and interpretations of the study authors including all themes, categories, theories, models, and similar. Therefore, all material labelled as results or findings by the authors as well as subsequent discussions and conclusions relating to CVADs will be extracted in full [9].

### Outcomes and prioritisation

The primary outcomes of interest will be any aspect of patients’ (or parent/guardians’) self-reported experiences or perspectives relating to PICC, Hickman, or Port devices. The data to be collected will include quantitative and qualitative materials, i.e. survey or questionnaire data, qualitative themes, and participant quotations.

For our present purposes, ‘patient perspectives’ will include opinions, attitudes, and evaluations (including acceptance and satisfaction). Patient experiences will include emotions, physical sensations (e.g. pain, discomfort), psychological factors (e.g. stress, mood), and pragmatic factors (e.g. routine activities).

Our analyses will prioritise any of the above as they relate to device acceptance or preference or to patient wellbeing or quality of life. Secondary prioritisation will be given to any of the above as they relate to device placement (insertion).

### Risk of bias in individual studies

Critical appraisal of methodological quality and risk of bias of included papers will be undertaken independently by two reviewers according to the Mixed Methods Appraisal Tool (MMAT) developed by Pluye and colleagues [10] for the appraisal of qualitative, quantitative, and mixed methods research. Following these guidelines, the reviewers will assess for all included studies whether (i) there are clear research questions and (ii) the data collected address the research question. The following assessments will then be made, depending on study type:

#### Qualitative studies

The reviewers will assess the appropriateness of data sources and analytical processes, the study’s consideration of context/setting, and the study’s consideration of researchers’ influence.

#### Randomised controlled trials

The reviewers will assess the study’s descriptions of randomisation and allocation concealment, the completeness of outcome data, and the level of drop-out rates.

#### Nonrandomised quantitative studies

The reviewers will assess the study’s minimisation of selection bias, use of appropriate measurement instruments, use of comparable groups across study conditions, and the completeness of outcome data.

#### Descriptive cross-sectional quantitative studies

The reviewers will assess the appropriateness of the study’s sampling strategy and the representativeness of the sample, use of appropriate measurement instruments, and acceptability of response rate.

### Data analysis and synthesis

Data will be analysed using a narrative approach, specifically thematic synthesis. Our analysis will comprise three phases. The first phase will be a thematic synthesis of the patient experience and perspective data extracted from the quantitative studies selected for inclusion in our review. We use inclusive definitions of patient experience and perspectives in this review. Our search strategy therefore includes a range of broad search terms relating to these phenomena. In addition, to our knowledge, there are no validated or standardised measures relating to patient experiences of CVADs in common usage. For instance, one such measure recently developed in France (specific to Port devices) [11] has to date been used in a single pilot study [12]. Consequently, we anticipate significant heterogeneity with regard to quantitative outcomes, and these data will not be quantitatively synthesised. The second phase of analysis will be a thematic synthesis of the qualitative studies selected for inclusion. The final third stage will be an aggregation of both sets of analysis. In the interests of transparency, detailed accounts of each stage of analysis will be recorded. The second phase of analysis will be a thematic synthesis of the patient experience and perspective data extracted from qualitative studies selected for inclusion. The final third stage will be an aggregation of both sets of analysis. In the interests of transparency, detailed accounts of each stage of analysis will be recorded.

### Analytic method

Thematic synthesis is an adaptation for the purpose of secondary data synthesis of ‘thematic analysis’ and provides a set of established methods and techniques for the identification and development of analytic themes in primary research data [9]. Thematic synthesis was selected for the purposes of this review for a several reasons [13]. First, it is well suited to our present objective of aggregating existing evidence and identifying patterns within data. Second, whilst it is most commonly associated with the synthesis of qualitative research outcomes, thematic synthesis is also used for the synthesis of quantitative research outcomes, particularly where there is heterogeneity in outcome variables and measurements. Finally, the process of thematic synthesis offers good transparency and outcomes are accessible.

Thematic synthesis involves three stages of analysis (all three stages will be applied to all included quantitative studies and repeated for all included qualitative studies). First, the data—here, those pertaining to patient experiences and perspectives—are coded. A coding frame will be developed comprising codes derived from the data. Coding will be carried out by the first reviewer and checked by a second reviewer. Disparities or discrepancies in coding will be resolved through discussion or in consultation with a third party if necessary; the coding frame will be adjusted accordingly.

In the second stage of analysis, similarities between codes will be identified. Codes will be grouped into ‘descriptive themes’ that capture and describe patterns in the data across studies. Each theme will be entered as columns into a table, and coded data from each study will illustrate the themes in rows, to facilitate comparison within and between studies, as part of the constant comparison analytic process [14, 15]. Memos will be used as part of the analysis process and may be included in the summary table where clarity may be required about the interpretation of a piece of evidence [16]. The aim of this table is to demonstrate themes with illustrative data and capture similarities and differences within the data where possible—that is, to show how themes are generated but also show divergence of findings in each theme, where it applies.

The third stage of thematic synthesis will involve the development of analytic themes. The purpose of this phase of analysis is to ‘go beyond’ the primary reported data by synthesising findings across studies and interpreting their meaning in relation to our review research questions. This will comprise the narrative component of our analysis, which will provide narrative descriptions of each theme.

Once thematic synthesis has been completed for quantitative studies and for qualitative studies, a final phase of analysis will compare both sets of results, and the three central venous access devices included in the review. This process will aggregate results, providing an overall account of current understandings of patient experiences and perspectives of CVADs in the context of anti-cancer treatment.

### Confidence in cumulative evidence

Confidence in discrete review findings will be assessed using the recently developed Confidence in the Evidence from Reviews of Qualitative research (CERQual) approach [17]. Assessment of confidence in a given review finding involves evaluating how likely it is that the finding represents a real phenomenon, i.e. genuine patient experiences of CVADs. This assessment will be based upon an evaluation of the following: (i) methodological limitations of the primary studies contributing to the finding, (ii) the relevance of the primary contributing studies with regard to the objectives of the systematic review, (iii) the coherence of the finding, and (iv) the adequacy of data supporting the finding.

For the purposes of this study, these evaluations will be conducted as follows:

(i) Methodological limitations of contributing studies: With reference to our prior critical appraisal of methodological quality of these studies (see above), we will evaluate the extent to which the primary studies contributing to the finding are generally of good methodological quality.
(ii) Relevance of contributing studies: We will evaluate the extent to which the primary studies contributing to the finding are similar in context and aims/objectives to the present review. For instance, studies carried out with the primary aim of investigating some aspect of patients’ experiences of CVADs in the context of cancer treatment will be considered highly relevant. Those in which findings relating to patient experiences of CVADs are secondary or incidental to an alternative aim will be considered to be of low relevance.
(iii) Coherence of finding: We will assess the extent to which the finding offers a credible explanation for the patterns it describes (e.g. tendencies, relationships between relevant factors). This will involve assessing the consistency or inconsistency of the pattern across various research contexts represented by studies included in the review and the ability of the finding to account for notable variations across contexts. Findings will be judged to be of low coherence if they describe inconsistent or contradictory patterns and where inconsistencies are unexplained.
(iv) Adequacy of supporting data: We will assess the extent to which a given finding is supported by substantial evidence defined both in terms of number of studies contributing to a given study and the quantitative robustness or qualitative richness (i.e. fullness and depth) of the data provided by these studies.

The above evaluations will necessarily entail subjective judgements and will therefore be carried out by two reviewers working in collaboration. A summary table will list for each review finding—primary contributing studies, evaluations of the above four domains, an overall confidence rating (high, moderate, low, or very low), and an explanation of the rating judgement.

## Discussion

The results section of the review will summarise the findings of (i) the thematic synthesis of quantitative studies, (ii) the thematic synthesis of qualitative studies, and (iii) the aggregation (comparison and juxtaposition) of both sets of findings.

The results will offer an account of current understandings of patient experiences and perspective regarding PICC, Hickman-type, and Port devices in the context of anti-cancer treatment. They will explore the extent to which and/or the ways in which these devices affect patients’ quality of life. Comparisons between experiences of different devices, or between especially positive and negative experiences, will be explored as appropriate and similarities and differences discussed and tabulated, if the data lend themselves to this. Not all relevant comparisons can be identified in advance, so other comparisons may become important. Finally, the results of this review will consider the gaps in the existing literature and of ways in which existing data can be used to support patients.

## Additional file

Additional file 1: PRISMA-P checklist. (DOCX 35 kb)

## Appendix

### Search strategy

#### Ovid MEDLINE(R) via Ovid 1946 to Week 2 2017

1. exp Vascular Access Devices/
2. Catheterization, Central Venous/
3. (central venous adj5 (catheter* or access)).tw.
4. percutaneous catheter*.tw.
5. (venous adj5 (catheter* or access)).tw.
6. vascular access.tw.
7. hickman*.tw.
8. ((perhiperally adj5 catheter*) or PICC*).tw.
9. (implant* adj5 catheter*).tw.
10. (venous adj5 Port*).tw.
11. (Portacath* or Port-a-cath*).tw.
12. (implant* adj5 Port*).tw.
13. (implant* adj5 reservoir*).tw.
14. (subcutaneous* adj5 Port*).tw.
15. (tunnel* adj5 catheter*).tw.
16. or/1-15
17. exp Neoplasms/
18. chemotherapy.tw.
19. (tumor* or tumour*).tw.
20. cancer*.tw.
21. malignan*.tw.
22. oncolog*.tw.
23. or/17-22
24. exp Patient Satisfaction/
25. (patient* adj5 experience*).tw.
26. (patient* adj5 perspective*).tw.
27. (patient* adj5 view*).tw.
28. (patient* adj5 attitude*).tw.
29. (patient* adj5 opinion*).tw.
30. (patient* adj5 satisf*).tw.
31. (patient* adj5 accept*).tw.
32. (patient* adj5 evaluat*).tw.
33. (patient* adj5 assess*).tw.
34. (patient* adj5 choice*).tw.
35. (patient* adj5 decision*).tw.
36. (patient* adj5 prefer*).tw.
37. (patient* adj5 (questionnaire* or survey*)).tw.
38. or/24-37
39. 16 and 23 and 38

## Abbreviations

CERQual: Confidence in the Evidence from Reviews of Qualitative research
CVAD: Central venous access device
MMAT: Mixed Methods Appraisal Tool for the appraisal of qualitative, quantitative, and mixed methods research
PICC: Peripherally inserted central catheter
PICO: Framework for the development of systematic literature searches. Acronym derived from key aspects of empirical studies: Population, Intervention, Comparison, Outcome
PRISMA-P: Preferred Reporting Items for Systematic Review and Meta-Analysis Protocols
SPIDER: Adaptation of PICO for the development of systematic searches of qualitative literature. Acronym derived from key aspects of empirical studies: Sample, Phenomenon of Interest, Design, Evaluation
